# Protective effect of crocin against d-galactose-induced aging in mice

**Published:** 2018

**Authors:** Elaheh Mohammadi, Soghra Mehri, Hasan Badie Bostan, Hossein Hosseinzadeh

**Affiliations:** 1 *Department of Pharmacodynamics and Toxicology, School of Pharmacy, Mashhad University of Medical Sciences, Mashhad, Iran*; 2 *Pharmaceutical Research Center, Department of Pharmacodynamics and Toxicology, School of Pharmacy, Mashhad University of Medical Sciences, Mashhad, Iran*

**Keywords:** D-galactose, Crocin, Crocus sativus, Aging, Oxidative stress

## Abstract

**Objective:**

Aging is a multifactorial phenomenon, which attribute to different diseases and abnormalities in living systems. Oxidative stress, which is an important factor in aging, exacerbates this process via different mechanisms. Crocin (CR), one of the active components of saffron showed strong antioxidant effects. In the present study, anti-aging property of crocin was investigated in mice.

**Materials and Methods:**

The model of aging was induced using administration of d-galactose (500 mg/kg, s. c.) for 42 days. Animals were treated with crocin (10, 20, 40 mg/kg, i.p.) during treatment with d-galactose. At the end of treatment, levels of malondialdehyde (MDA) as a lipid peroxidation marker and glutathione content (GSH) in the liver and brain were measured. Also, biochemical factors including liver enzymes (ALT and AST), male sex hormones including testosterone and dehydroepiandrosterone-sulfate (DHEA-SO_4_) and pro-inflammatory markers such as tumor necrosis factor -α (TNF-α) and interlukine-6 (IL-6) in serum, were evaluated.

**Results:**

Administration of d-galactose led to induction of lipid peroxidation in liver and brain tissues, as well as elevation of AST, ALT, and pro-inflammatory cytokines and reduction of male sex hormones levels in serum. Interestingly, treatment of animals with crocin (10, 20 and 40 mg/kg) diminished lipid peroxidation in the liver and brain tissues while elevated GSH content. Also, a decline in serum levels of TNF-α and IL-6 and an elevation of male sex hormones were observed following treatment with crocin.

**Conclusion:**

Administration of crocin reduced d-galactose-induced aging in mice through inhibition of oxidative stress, reduction of inflammation and elevation of sex hormones.

## Introduction

Aging as a natural biological process results in the dysfunction of normal cells. As a causative phenomenon, aging is correlated with various chronic disorders such as cancer, cardiovascular and Parkinson’s diseases (Zhong et al., 2009 ▶; Wang et al., 2012 ▶). Aging is responsible for more than 50% of Alzheimer’s disease occurrence. Among the all hypotheses, free radical theory of aging is well-known and the first time, it was reported by Harman in 1956 (Hsieh et al., 2009 ▶; Wang et al., 2012 ▶). This theory states that production of oxygen-derived free radicals can lead to tissue and cell oxidative damage, finally resulting in aging and cell death (Hsieh, et al., 2009 ▶). So, it has been thought that oxidative damage is a key-factor in aging phenomenon (Lu et al., 2006 ▶). Detrimental effects of reactive oxygen species (ROS) on cell macromolecules such as proteins, DNA, and cell membrane lipids have been observed (Lan et al., 2012 ▶). It was demonstrated that ROS production can cause mutations in mitochondrial DNA, resulting in malfunction of respiratory chain in this organelle. Moreover, it was proposed that this event could induce ROS production which leads to a vicious circle (Ruan et al., 2013 ▶).

Parallel to the increase in elderly population, anti-aging approaches have become an important public issue. Fortunately, herbal medicines have opened new horizons and attracted great attention as potential agents for development of new anti-aging drugs (Wang et al., 2012 ▶). Herbal plants, as the main source of natural antioxidants, are known to be beneficial for biological systems by protecting them against oxidative damages.

Saffron or *Crocus sativus* L. (family: Iridaceae), has a special place in Islamic herbal medicine (Mollazadeh et al., 2015 ▶). Recently, several studies have focused on pharmacological activities of saffron and its main components. Crocin is one of the active metabolites of saffron which is responsible for its red color (Alavizadeh and Hosseinzadeh, 2014 ▶). Many pharmacological studies have demonstrated that crocin can be used as a new treatment, due to its antitumor (Alavizadeh and Hosseinzadeh 2014 ▶), antioxidant (Jauslin ML et al., 2007 ▶), radical-scavenging (Razavi and Hosseinzadeh, 2013 ▶), cardioprotective (Razavi and Hosseinzadeh, 2013 ▶), spatial cognitive ability improving (Hosseinzadeh et al., 2012 ▶), anti-inflammatory (Nam et al., 2010 ▶; Li et al., 2015 ▶), and hepatoprotective effects (Bahashwan et al., 2015 ▶) as well as having protective properties against DNA damage (Hosseinzadeh et al., 2008 ▶).

Crocin significantly reduced LPS (Lipopolysaccharides)-induced overexpression of pro-inflammatory factors including interleukin-1β (IL-1β), (TNF)-α and IL-6 in a concentration-dependent manner (Li K et al., 2015 ▶) .

D-galactose is a monosaccharide sugar that can be metabolized if administered within normal concentration range. It has been shown that over-supply of d-galactose leads to disruption of metabolic pathway. D-galactose is changed to aldose and hydrogen peroxide by galactose oxidase enzyme. In addition, it is converted to galactitol which accumulates in the cell and leads to osmotic stress and production of ROS (Kumar et al., 2011 ▶).

Similar conversion occurs in natural aging in the body and it leads to neurological impairments, reduced activity of antioxidant enzymes, atherosclerosis and arthritis (Zhang et al., 2009 ▶; Ghanbari et al., 2012 ▶; Haider et al., 2015 ▶). Hence, d-galactose is used for induction of natural aging models, *in vivo* (Hsieh et al., 2009 ▶).

Different changes in the immune system, known as immunosenescence and elevation in the production of cytokines by adipose tissue, have been reported in aging phenomenon (Michaud et al., 2013 ▶) . 

Overall, oxidative stress and inflammation have important role in aging process. As mentioned above, crocin has shown antioxidant and anti-inflammatory properties in different conditions. Therefore, the current study was designed to evaluate anti-aging effect of crocin on d-galactose-induced aging in mice through determination of lipid peroxidation and GSH content as well as measuring inflammatory markers and sex hormone levels. 

## Materials and Methods

**Chemicals**

Malondialdehyde tetrabutylammonium, DTNB (5- 5-dithiobis-(2-nitrobenzoic acid)) and d-galactose were prepared from Sigma. Mouse TNF-α ELISA kit and Mouse IL-6 ELISA kit were purchased from eBioscience.

**Crocin preparation**

Stigmas of *Crocus sativus* L. were obtained from Novin Saffron (collected from Ghaen, Khorasan province, Northeast of Iran) and analyzed according to the ISO/TS 3632-2. Extraction and purification of crocin was done according to the method which was described previously (Hadizadeh et al., 2010 ▶). The purity of crocin was 97%.

**Animals**

Male Razi mice with the weight of 25–27 g at the start of the experiment, were obtained from Faculty of Pharmacy, Mashhad University of Medical Sciences, Mashhad, Iran. Animals were kept under controlled conditions (with 12 hr:12 hr light/dark cycle at 21 ± 2 ºC). They had free access to food and water. All animal experiments were done according to Mashhad University of Medical Sciences, Ethics Committee Acts. 

**Experimental design**

To induce aging, animals were treated with d-galactose (500 mg/kg, s.c.) for 42 days.

This daily dose and corresponding route of exposure have been well known for induction of aging (Ghanbari et al., 2012 ▶; Jin S-l and Yin Y-g 2012 ▶).

In our study, rats were randomly divided into 6 groups (n=10 in each group) and treated as follows: 

1- Control, normal saline 

2- D-galactose (500 mg/kg, s.c.) for 42 days

3- D-galactose (500 mg/kg, s.c.) for 42 days + crocin (10 mg/kg, i.p.) (Mehri et al., 2015 ▶)

4- D-galactose (500 mg/kg, s.c.) for 42 days + crocin (20 mg/kg, i.p.) (Mehri et al., 2015 ▶)

5- D-galactose (500 mg/kg, s.c.) for 42 days + crocin (40 mg/kg, i.p.) (Mehri et al., 2015 ▶)

6- Crocin (40 mg/kg, i.p.) 

Intraperitoneal administration of crocin was done on a daily basis.

At the end of the treatment, blood samples were collected under anesthesia using cardiac puncture and kept for 1 hr. Serum samples were separated by centrifuging blood at 4°C for 10 min. Liver and brain tissues were removed, snap-frozen in liquid nitrogen and transferred to -80 ºC freezers until use.

**Measurement of lipid peroxidation (Tiobarbituric acid reactive species)**

The final product of lipid peroxidation is malondialdehyde (MDA) which reacts with TBA resulting in production of a pink color complex which has a maximum absorbance at 532 nm (Uchiyama and Mihara 1978 ▶). For this assay, brain or liver tissue homogenate 10% in KCl was prepared. Then, 3 ml phosphoric acid (1%) and 1ml TBA (0.6%) were added to 0.5 ml tissue homogenate 10% and the mixture was heated for 45 min in a boiling-water bath. After cooling, 4 ml n-butanal was added to this mixture and it was mixed for 1 min. Next, the samples were centrifuged at 3000 *g* for 10 min. The absorbance of organic layer was measured at 532 nm. MDA quantity was reported as nmol/g tissue.

**Measurement of reduced glutathione (GSH) content**

GSH content was assessed according to Moron et al (1979) ▶ procedure with some changes (Moron et al., 1979 ▶). Briefly, 500 µl of 10% tricolor acetic acid (TCA) was added to 500 µl of homogenated tissue. Subsequently, this mixture was vortexed and centrifuged at 2500 *g* for 10 min. Then, the supernatant was separated and mixed with 2 ml phosphate buffer (pH: 8) and 500 µl DTNB (5- 5-dithiobis-(2-nitrobenzoic acid)). The absorbance was determined at 412 nm by a spectrophotometer (Jenway 6105 UV/VIS, UK). GSH levels were calculated based on a standard curve prepared using commercially available GSH. GSH levels were reported as nmol/g tissue.

**Measurement of testosterone and dehydroepiandrosterone- sulfate (DHEA-SO**_4_ **)**

Level of sex hormones including testosterone and dehydroepiandrosterone- sulfate were measured according to kit protocols and data were expressed as ng/mL (for testosterone) and ng/dL (for DHEA-SO_4_). 

**Measurement of ALT and AST**

The levels of these enzymes were determined by Mindary auto analyzer (BS 800) according to kits’ protocols and data were expressed as IU/L. 

**Measurement of TNF-α and IL-6**

TNF-αand IL-6 levels were quantitatively measured in serum using TNF-α and IL-6 ELISA kits (eBioscience). The absorbance of the final colored product was recorded at 450 nm as the primary wave length and at 650 nm as the reference wave length. TNF-α and IL-6 levels were expressed as pg/mL.

**Statistical analyses**

All statistical analyses were performed using one-way ANOVA followed by Tukey-Kramer test for multiple comparisons to evaluate the discrepancy of the data. P-values less than 0.05 were considered statistically significant.

## Results

**Effect of crocin on d-galactose-induced lipid peroxidation in mice**

**Figure 1:**
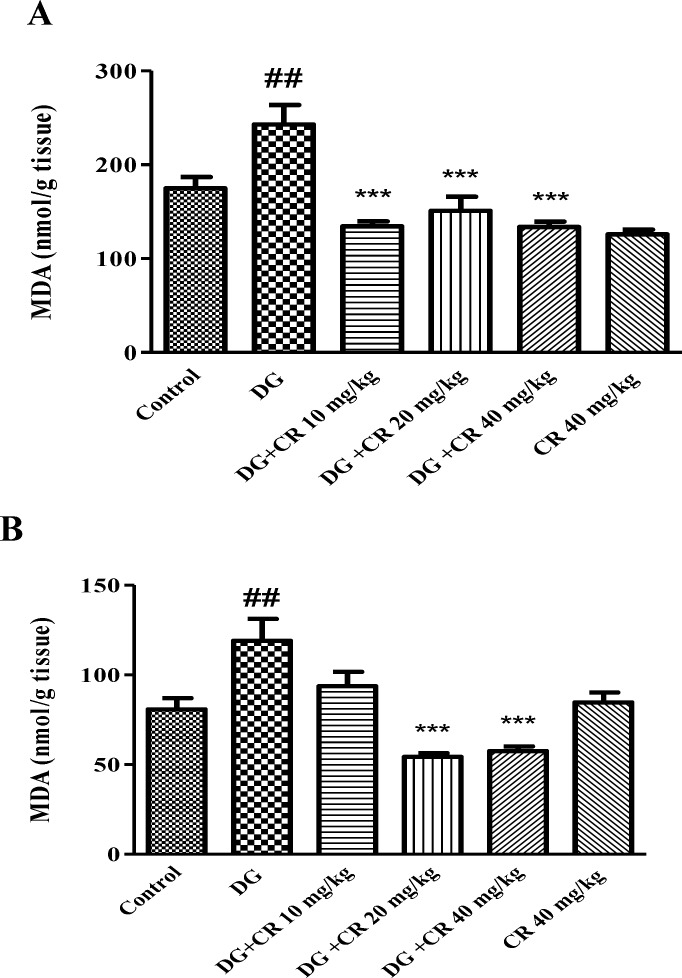
Effect of crocin on lipid peroxidation induced by d-galactose in the brain (A) and liver (B). Data are presented as mean ± SD (n=7). ^# #^ p<0.01 vs control and ^***^ p<0.001 vs d-galastose-treated animals. DG (d-galactose); CR (crocin

Administration of d-galactose for 42 days markedly induced lipid peroxidation in brain and liver tissues in comparison to control group (p<0.01). Nevertheless, treatment with crocin (20 and 40 mg/kg) significantly recovered lipid peroxidation (p<0.001) (Figures 1A and 1B).

**Effect of crocin on d-galactose-induced GSH depletion in mice**

**Figure 2:**
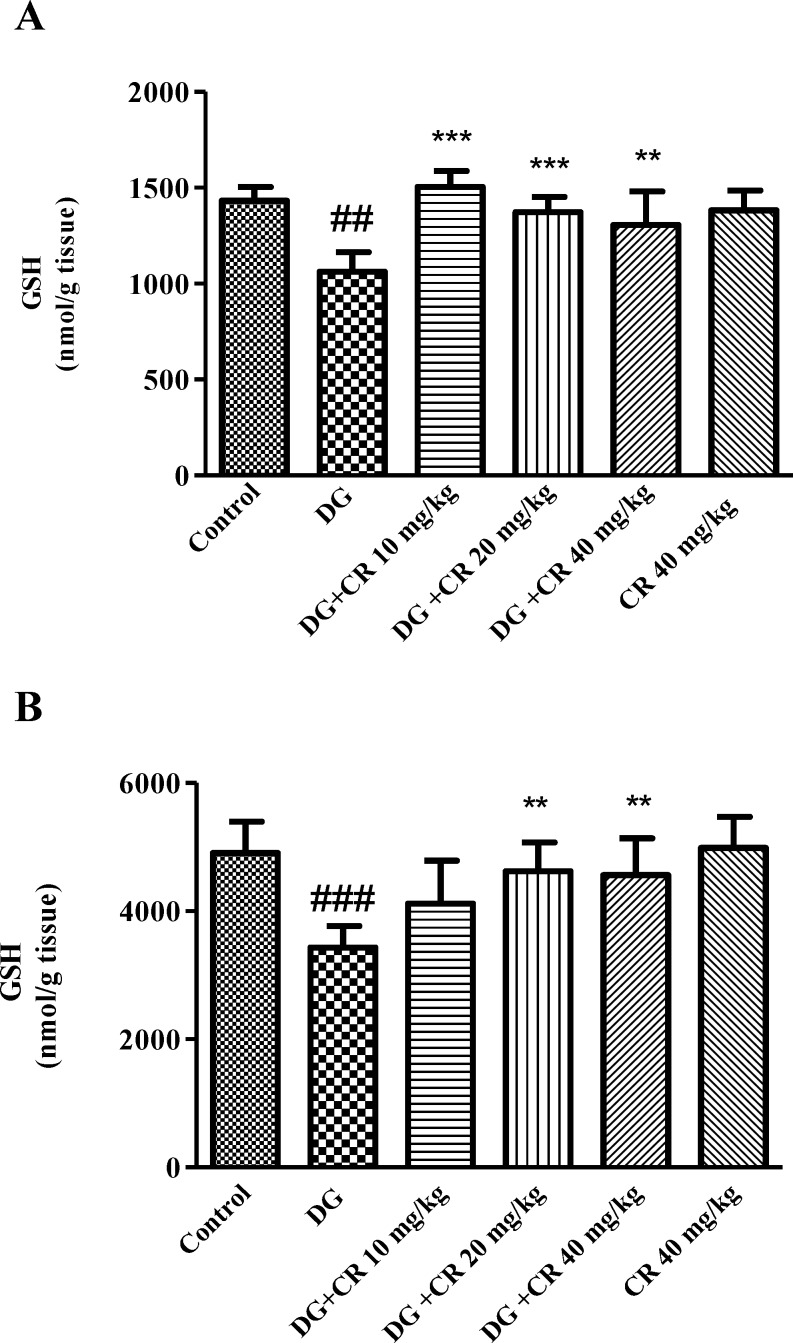
Effect of crocin on brain (A) and liver (B) GSH content following treatment with d-galactose. Data are presented as mean ± SD (n=7). ^##^ p<0.01 and ^###^ p<0.001 vs control,

^**^ p<0.01 and ^***^ p<0.001 vs d-galactose-treated animals. DG (d-galactose); CR (crocin).

As shown in Figure 2A, significant reduction in GSH content was observed following exposure to d-galactose when compared to control group (p*<*0.001). Treatment with 10, 20 and 40 mg/kg crocin significantly increased GSH content as compared to d-galactose-treated animals. 

Additionally, there was a significant reduction in GSH content in liver tissue (p<0.001 vs control) while crocin (20 and 40 mg/kg) markedly elevated GSH level in comparison to d-galactose group (p<0.01) (Figure 2B).

**Effect of crocin on serum levels of ALT and AST in mice with d-galactose-induced aging**

As indicated in Table. 1, d-galactose administration caused significant elevation in ALT and AST levels (p<0.01). Elevated level of ALT and AST enzymes was recovered in animals that received 20 mg/kg crocin (p*<*0.001 for ALT and p*<*0.01 for AST).

**Effect of crocin on sex hormone levels in mice serum of d-galactose-induced aged mice**

Treatment with d-galactose significantly diminished testosterone and DHEA-SO_4 _levels in comparison to control (p<0.01 and p<0.05 respectively). As shown in Table. 1, following exposure to crocin (10 and 20 mg/kg) a significant enhancement was observed in both levels of testosterone and DHEA-SO_4_ in aged mice. 

**Effect of crocin on level of TNF-α and IL-6 in d-galactose-induced aged mice**

**Figure 3:**
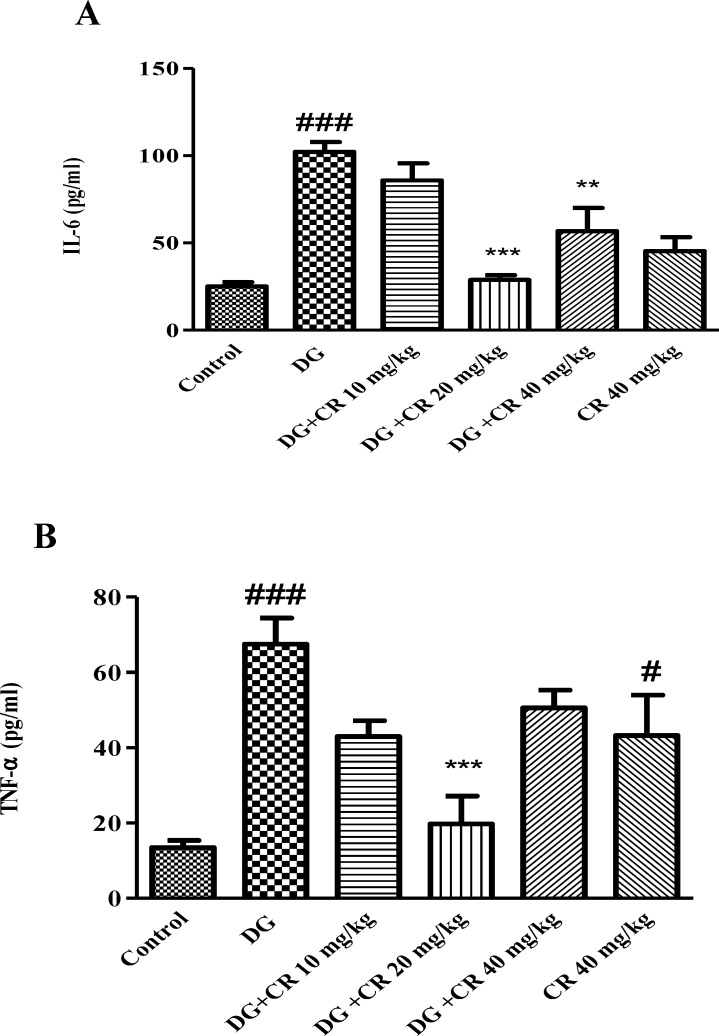
Effect of crocin on IL-6 (A) and TNF-α (B) levels in mice serum following induction of aging by d-galactose. Data are presented as mean ± SD (n=5).^#^ p<0.05,^ ###^ p<0.001vs control; ^**^p<0.01

As shown in Figure. 3A and B, increased level of IL-6 and TNF-α were observed when animal exposed to d-galactose (p<0.001 vs control). Administration of crocin 20 mg/kg markedly diminished level of inflammatory markers in comparison to d-galactose treated group (p<0.001). 

and ^***^p<0.001 vs d-galactose-treated animals. DG (d-galactose); CR (crocin).

**Table 1:** Effect of crocin on ALT, AST and sex hormones levels in mice serum following induction of aging by d-galastose

**Test** **Group**	**ALT (IU/mL)**	**AST (IU/mL)**	**Testosterone (ng/dl)**	**DHEA-SO**_4_ **(ng/ml)**
**Control**	135.87±21.25	251.16±11.05	15±2.68	40.28±3.2
**D-galactose (DG)**	176.25±14.55##	335±32.66##	5.6±3.32##	26±9.29#
**DG+ CR 10 mg/kg**	152.57±11.41	297.4±32.63	24.6±8.1***	43.2±8.31**
**DG+ CR 20 mg/kg**	128.2±15***	247.5±32.57**	30.16±4.5***	40.5±4.12*
**DG+ CR 40 mg/kg**	144.28±25.28*	307.5±53.99	11.16±3.53	27±6.21
**CR 40 mg/kg**	149.6±14.68	242.2±39.97	18.8±6.45	28.5±10.87

Data are expressed as mean ± SD (n=7).

# p<0.05 and

## p<0.01 vs control;

* p<0.05,

** p<0.01 and

*** p<0.001 vs d-galactose-treated animals. DG (d-galactose); CR (crocin) AST (aspartate aminotransferase); ALT (alanine amino transferase); DHEA-SO_4_ (dehydroepiandrosterone- sulfate).

## Discussion

The anti-aging effect of crocin against d-galactose-induced aging was evaluated in mice. In agreement with other studies, administration of d-galactose (s. c.) for 42 days significantly elevated oxidative stress in the liver and brain. Additionally, a considerable enhancement in the level of biochemical markers including ALT, AST, IL-6 and TNF-α in aged animals was observed. Treatment with crocin (10, 20 and 40 mg/kg) reversed aging consequences in mice through reduction of lipid peroxidation, improvement of liver function, inhibition of inflammation and elevation of sex hormones.

D-galactose, a kind of reducing sugar, is responsible for stimulating ROS production and disturbing carbohydrate metabolism finally resulting in oxidative stress in the body. It has been found that long-term exposure to d-galactose could be used for induction of an aging model in animals. It was demonstrated that exposure to d-galactose potentially induce age-related diseases such as neurological impairments and antioxidant fragility which are very similar to what happens in natural aging (Ghanbari et al., 2012 ▶). Oxidative damages are one of the important factors which could contribute to aging phenomenon. MDA, a lipid peroxidation by-product, is known as a good index of oxidation status and it is even regarded as the marker of aging (Rikans and Hornbrook 1997 ▶). MDA could bind to free amino groups of proteins resulting in MDA-modified protein adducts. 

In the current study, treatment of animals with d-galactose significantly reduced GSH content with parallel enhancement of MDA level in both brain and liver tissues which accordance with before reports (Zhang et al., 2009 ▶; Zhen et al., 2016 ▶). 

As our result showed, crocin alleviated liver and brain oxidative injury through increasing GSH content and decreasing MDA level. The best effects of crocin in elevation of GSH content and inhibition of lipid peroxidation in both brain and liver tissues were observed at 20 and 40 mg/kg doses. Moreover, hepatoprotective and nuroprotective effects of crocin have been demonstrated in other studies. Crocin showed protective effects against reperfusion-induced oxidative/ nitrosative injury following global cerebral ischemia in mice (Zheng et al., 2007 ▶). Also, crocin significantly inhibited acrylamide-induced neurotoxicity in Wistar rats through antioxidant activity (Mehri et al., 2015 ▶). Treatment of animals with crocin reduced diazinon-induced hepatotoxicity specially via inhibition of apoptosis (Lari et al., 2015 ▶). 

Liver enzymes including ALT and AST are indicative of liver function. Increase in these markers are one of the signs which show liver injury (Anderson et al., 2000 ▶). There are numerous studies that have shown that ALT and AST increase significantly after administration of hepatotoxic agents including diazinon and carbon tetrachloride to animals (Hariri et al., 2010 ▶;Jnaneshwari et al., 2013 ▶; Bahashwan et al., 2015 ▶). Nevertheless, treatment with crocin showed remarkable effects on alleviating this damage (Hariri et al., 2010 ▶; Bahashwan et al., 2015 ▶). As observed, our result is consistent with these findings and crocin 20 mg/kg markedly reduced the level of ALT and AST enzymes. 

Lack of homeostasis in inflammatory cytokines (i.e. TNF-α, IL-1 and IL-6) production, could lead to immune system dysfunction and tissue inflammation. Hyperactivity of B-cells and T-cells results in increased level of these cytokines in the body (Umare et al., 2014 ▶).

 IL-6 is a pro- inflammatory cytokine which regulates several cellular processes such as erythropoiesis and neuronal cell degeneration. TNF-α plays important roles in immunoregulation, inflammation and programmed cell death (Umare et al., 2014 ▶). It has been reported that the level of pro-inflammatory cytokines including TNF-α and IL-6 elevated in aging (Michaud et al., 2013 ▶). Administration of d-galactose to male BALB/c mice significantly elevated the level of pro-inflammatory markers such as TNF-α, IL-β and IL-6 in blood samples (Ghanbari et al., 2012 ▶; Mohammadirad et al., 2013 ▶). In another study, significant elevation in the plasma level of TNF-α and IL-β was observed following treatment of rats with d-galactose (Hadzi-Petrushev et al., 2014 ▶)

Crocin, a carotenoid derived from saffron, showed anti-inflammatory propertied in different studies (Nam et al., 2010 ▶; Li et al., 2015 ▶). Also, after crocin treatment, a decrease in the level of TNF-α and IL-6 has been reported. In accordance with these findings, our results showed a significant decrease in TNF-α and IL-6 levels in aged mice which received crocin.

 Interestingly, it has been found that there is an inverse relationship between testosterone concentration and pro-inflammatory cytokines level in aging. These factors (i.e. IL-6, TNF-α and IL-1β) inhibit the secretion of testosterone hormone by affecting the hypothalamic-pituitary and testicular component of gonadal axis. Conversely, supply of testosterone could decrease inflammatory cytokines levels (Maggio et al., 2005 ▶). The results of the current study exhibited increased level of pro-inflammatory cytokines with parallel decrease of sex hormone in aged mice.

Additionally, previous studies showed that the level of sex hormones diminished in aged mice while treatment with protective agents restored it (Ghanbari et al., 2012 ▶; Mohammadirad et al., 2013 ▶). 

Nazem et al, (2009) ▶ showed that saffron administration to male BALB/c elevated the level of testosterone, LH and FSH hormones in serum. This might possibly be due to the effect of saffron which reduced hypophyseal–hypothalamus sensitivity to testosterone feedback control on LH secretion (Asadi et al.,2014 ▶). Furthermore, in our previous examinations, results exhibited that crocin and saffron increased mounting, intromission and erection in rats (Shamsa et al., 2009 ▶). In other reports safety of crocin has been mentioned (Hosseinzadeh et al., 2013 ▶; Mohamadpour et al., 2013 ▶). 

Interestingly, in our study elevated level of testosterone and DHEA-SO_4 _following treatment with crocin 20 mg/kg in aged mice was observed.

Result of the present study showed the anti-aging effect of crocin in a mice model of aging induced by d-galactose. Elevation of GSH content, reduction of lipid peroxidation and inhibition of inflammation might be the underlying mechanisms.
